# SLEEP DISTURBANCES AND PAIN ON DEPRESSION IN OLDER ADULTS WITH AND WITHOUT ALZHEIMER’S DISEASE

**DOI:** 10.1093/geroni/igae098.3719

**Published:** 2024-12-31

**Authors:** YingHua Wu, Benjamin Hougaard, Bárbara Gómez Cáceres, Jamie Kiefer, Rowena Gomez

**Affiliations:** Palo Alto University, Watsonville, California, United States; Palo Alto University, Florence, Wisconsin, United States; Palo Alto University, Palo Alto, California, United States; Palo Alto University, Palo Alto, California, United States; Palo Alto University, Palo Alto, California, United States

## Abstract

Pain is associated with depression and sleep disturbance in older adults (OAs), including those with Alzheimer’s disease (AD; Cao et al., 2019; Tsai et al., 2021). However, limited research examines how various sleep disturbances moderate the relationship between pain and depression in OAs and those with and without AD. From the Health and Retirement Study, the sample included 15,512 OAs without AD (59% female; Mage = 69.28; SDage = 10.81) and 211 OAs with Alzheimer’s disease (64.9% female; Mage = 81.15; SDage = 9.82). Depression was measured based on 14 questions about depressive symptoms corresponding with criteria A of major depressive episode. Pain was assessed by two survey questions about present pain and severity. The severity of different sleep disturbances was also assessed. Custom ANOVAs indicated that trouble waking up too early (p <.001), trouble falling asleep (p <.001), trouble waking up during the night (p <.001), and feeling rested in the morning (p <.001) significantly moderated the effect of pain on depression severity in OAs without AD. However, the moderation was not significant in OAs with AD, though there was a main effect of trouble falling asleep, feeling rested in the morning, and pain on depression. Interventions aimed at improving specific aspects of sleep may help mitigate the effects of pain on depression severity in OAs without AD. The absence of this moderation in OAs with AD still suggests the impact of sleep disturbance and pain on depression.

